# Effect of the Novel Polysaccharide PolyGlycopleX^®^ on Short-Chain Fatty Acid Production in a Computer-Controlled *in Vitro* Model of the Human Large Intestine

**DOI:** 10.3390/nu6031115

**Published:** 2014-03-14

**Authors:** Raylene A. Reimer, Annet J. H. Maathuis, Koen Venema, Michael R. Lyon, Roland J. Gahler, Simon Wood

**Affiliations:** 1 Faculty of Kinesiology and Department of Biochemistry & Molecular Biology, University of Calgary, Calgary, AB T2N 1N4, Canada; 2 TNO, Healthy Living, P.O. Box 360, Zeist, AJ 3700, The Netherlands; E-Mails: annet.maathuis@tno.nl (A.J.H.M.); koen.venema@outlook.com (K.V.); 3 Canadian Centre for Functional Medicine, 1552 United Boulevard, Coquitlam, BC V3K 6Y2, Canada; E-Mail: doctorlyon@shaw.ca; 4 University of British Columbia, Food, Nutrition and Health Program, Vancouver, BC V6P 2G9, Canada; E-Mail: simonwood@shaw.ca; 5 Factors Group R & D, 3655 Bonneville Place, Burnaby, BC V3N 3S9, Canada; E-Mail: rgahler@naturalfactors.com; 6 InovoBiologic Inc., 104-1240 Kensington Road NW, Suite 409, Calgary, AB T2N 4X7, Canada; 7 School of Public Health, Faculty of Health Sciences, Curtin University, Perth, WA 6845, Australia

**Keywords:** dietary fiber, functional fiber, microbial fermentation, polysaccharide, propionate

## Abstract

Many of the health benefits associated with dietary fiber are attributed to their fermentation by microbiota and production of short chain fatty acids (SCFA). The aim of this study was to investigate the fermentability of the functional fiber PolyGlyopleX^®^ (PGX^®^) *in vitro*. A validated dynamic, computer-controlled *in vitro* system simulating the conditions in the proximal large intestine (TIM-2) was used. Sodium hydroxide (NaOH) consumption in the system was used as an indicator of fermentability and SCFA and branched chain fatty acids (BCFA) production was determined. NaOH consumption was significantly higher for Fructooligosaccharide (FOS) than PGX, which was higher than cellulose (*p* = 0.002). At 32, 48 and 72 h, acetate and butyrate production were higher for FOS and PGX *versus* cellulose. Propionate production was higher for PGX than cellulose at 32, 48, 56 and 72 h and higher than FOS at 72 h (*p* = 0.014). Total BCFA production was lower for FOS compared to cellulose, whereas production with PGX was lower than for cellulose at 72 h. In conclusion, PGX is fermented by the colonic microbiota which appeared to adapt to the substrate over time. The greater propionate production for PGX may explain part of the cholesterol-lowering properties of PGX seen in rodents and humans.

## 1. Introduction

Dietary fiber and resistant starches are substrates for bacterial fermentation in the human gastrointestinal tract that result in production of short chain fatty acids (SCFA), predominantly acetate, propionate and butyrate [1]. The interest in SCFA has grown given the mounting evidence for their role in colonic health and metabolic diseases, including irritable bowel syndrome, inflammatory bowel disease, obesity, cardiovascular disease and cancer [1]. Beyond the non-digestible carbohydrates naturally present in foods, there is also potential for novel fibers, those that are extracted and modified through physical, chemical or enzymatic means [2], to influence the production of SCFA. PolyGlycopleX^®^ (PGX^®^) is a novel polysaccharide that is manufactured by complexing three soluble viscous polysaccharides. While it is believed that this new complex should be fermented by the intestinal microbiota, this hypothesis was tested experimentally in this study.

We have previously demonstrated that PGX^®^ was associated with higher faecal total SCFA concentrations in healthy adults consuming PGX^®^ *versus* placebo for three weeks [3]. One drawback of measuring faecal SCFA, however, is difficulty in determining their exact production due to the substantial (up to 95%) absorption and metabolism of SCFA in the gut [4]. In contrast, SCFA production can be accurately measured in a computer controlled model of the proximal large intestine [5,6,7]. The *in vitro* TIM-2 system is a multi-compartmental and dynamic laboratory system, which simulates to a high degree the colon, including a complex, high density, metabolically active, anaerobic microbiota of human origin. This model has been validated with regards to the number and ratio of the various micro-organisms which are similar in composition and metabolic activity with that of the human colon as well as the production of metabolites, such as SCFA, branched short chain fatty acids (BCFA; *iso*-butyrate and *iso*-valerate), ammonia and phenolic compounds [5,6,7].

The ability to measure BCFA in addition to SCFA is important given the unique origin and fate of these fermentative products. While carbohydrates are fermented by saccharolytic bacteria and produce SCFA, H_2_ and CO_2_ [8], proteins and amino acids can also be fermented in the gut by proteolytic bacteria to yield BCFA, H_2_, CO_2_, CH_4_, phenols and amines [9]. Many products of protein fermentation have toxic effects [10].

Given the link between colonic health and reduced risk of metabolic disease and the role of SCFA in providing that protection, our objective was to determine the fermentation potential of the novel viscous polysaccharide, PGX^®^, using the *in vitro* TIM-2 system [5]. Fructooligosaccharide (FOS), a highly fermentable prebiotic fiber [9], was used as the positive control and cellulose, an insoluble fiber [11] as the negative control.

## 2. Experimental Section

### 2.1. Test and Control Fiber

The experimental fibers were: (1) Positive control: Fructo-oligosaccharide from chicory (Sigma Aldrich Chemie BV, Zwijndrecht, The Netherlands); (2) Negative control: Cellulose (VWR International BV, Roden, The Netherlands); (3) Test fiber: PolyGlycopleX^®^ (PGX^®^), a soluble high viscosity polysaccharide (HVP) manufactured by a proprietary process (EnviroSimplex^®^) from three dietary fibers; konjac (glucomannan), sodium alginate and xanthan gum (PGX^®^: α-d-glucurono-α-d-manno-β-d-manno-β-d-gluco, α-l-gulurono-β-d-mannurono, β-d-gluco-β-d-mannan; InovoBiologic Inc., Calgary, Canada). The carbohydrate properties of PGX have been previously described [12,13,14] and show that an interaction between a konjac glucomannan–xanthan gum complex and sodium alginate forms a new, ternary complex.

### 2.2. Intestinal Conditions of TIM-2

The conditions of the large intestine were reproduced in the TNO dynamic computer-controlled *in vitro* intestinal model, TIM-2. Details of the *in vitro* model have been previously published [5,7,15]. The following standardized conditions were simulated in the TIM-2 system: body temperature, pH in the lumen, composition and rate of secretion, delivery of a pre-digested substrate from the “ileum” (SIEM; Standard Ileal Efflux Medium), mixing and transport of the intestinal contents, absorption of water, presence of a complex, high density, metabolically active, anaerobic microbiota of human origin and absorption of microbial metabolites via a semi permeable membrane inside the colon model. The SIEM simulates material reaching the colon in humans. For experiments in the TIM-2, SIEM was modified slightly from the medium described by Gibson *et al.* [16], mainly concerning the concentration of carbohydrates, pepton, casein and Tween 80 [5,7]. SIEM contained the following components (g/L): 9.0 pectin, 9.0 xylan, 9.0 arabinogalactan, 9.0 amylopectin, 43.7 casein, 74.6 starch, 31.5 Tween 80, 43.7 bactopepton, 0.7 ox-bile, 4.7 g K_2_HPO_4_·3H2O, 8.4 g NaCl, 0.009 g FeSO_4_·7H_2_O, 0.7 g MgSO_4_·7H_2_O, 0.8 g CaCl_2_·2H_2_O, 0.02 g haemin and 0.3 g cysteine HCl, plus 1.5 mL of a vitamin mixture containing (per litre): 1 mg menadione, 2 mg d-biotin, 0.5 mg vitamin B12, 10 mg pantothenate, 5 mg nicotinamide, 5 mg *p*-aminobenzoic acid and 4 mg thiamine. The pH was adjusted to 5.8.

Prior to the performance of each experiment, the secretion fluids and dialysis solutions were prepared fresh, the pH electrodes calibrated, new membrane units installed and the system inoculated (one day before the start of the test period) with a microbiota of human origin (adults on Western type of diet as described in detail in [15]). After an overnight adaptation period in which the microbiota adapted to the model conditions, the test period started.

### 2.3. Addition of Test and Control Fibers

During the test period the TIM-2 units were continuously fed with SIEM, but without the standard carbohydrates. The test- and control products were added to TIM-2 during the day on all three test-days in an 8 h period (Table 1). The added dose was normalized on the maximum dose of PGX^®^ which could be added to TIM-2 during the day (4.0 g, 2.5 g and 3.5 g on each of the 3 consecutive days). The control products were added in at these doses in a similar timeframe as the test product. PGX^®^ was added to the lumen of TIM-2 (120 mL) during the day. Because of its viscosity, PGX^®^ was added to a large volume (300 mL) of dialysate before addition to TIM-2. The controls were added to TIM-2 in the same way. A total of 3 independent 72 h runs were made with PGX and 2 runs each for FOS and cellulose.

**Table 1:** Composition of media with control and test fibers.

Medium Component	Day 1/Day 2/Day 3	Day 1/Day 2/Day 3	Day 1/Day 2/Day 3
Casein	2.6 g	2.6 g	2.6 g
Tween 80	1.9 g	1.9 g	1.9 g
Bactopepton	2.6 g	2.6 g	2.6 g
Ox-bile	42 mg	42 mg	42 mg
K_2_HPO_4_·3H_2_O	282 mg	282 mg	282 mg
NaCl	0.5 g	0.5 g	0.5 g
FeSO_4_·7H_2_O	0.54 mg	0.54 mg	0.54 mg
MgSO_4_·7H_2_O	42 mg	42 mg	42 mg
CaCl_2_·2H_2_O	48 mg	48 mg	48 mg
Haemin	1.2 mg	1.2 mg	1.2 mg
Cysteine·HCl	18 mg	18 mg	18 mg
Vitamins *	90 mg	90 mg	90 mg
FOS	4.0 g/2.5 g/3.5 g		
PGX^®^		4.0 g/2.5 g/3.5 g	
Cellulose			4.0 g/2.5 g/3.5 g

* The vitamin formulation contained (mg/L): menadione, 1; biotin, 2; vitamin B_12_, 0.5; pantothenate, 10; nicotinamide, 5; *para*-aminobenzoic acid, 5; thiamine, 4.

### 2.4. Sampling from TIM-2

Lumen samples were collected in duplicate at the start of the test period and after 8, 24, 32, 48, 56 and 72 h. Total volumes of the dialysis liquid were measured and samples were collected at the start of the test period and after 8, 24, 32, 48, 56 and 72 h. Samples were stored at <−18 °C.

### 2.5. Fermentation and pH

Fermentation of non-digestible carbohydrates by the microbiota produces SCFA and lactate which cause the lumen to acidify. During the TIM-2 experiments pH was regulated to pH 5.8 by sodium hydroxide (NaOH) secretion. Sodium hydroxide usage during the experiments therefore indicated the ability of the microbiota to ferment the carbohydrates added to the TIM-2 system.

### 2.6. Short-Chain and Branched Chain Fatty Acid Analysis

SCFA (acetate, propionate, *n*-butyrate) and BCFA (*iso*-butyrate and *iso*-valerate) analyses were performed as described before [7]. In brief, samples were centrifuged (~12,000× *g*, 5 min) and a mixture of formic acid (20%), methanol and 2-ethyl butyric acid (internal standard, 2 mg/mL in methanol) was added to the clear supernatant. A 0.5 µL sample was injected on a GC-column (Stabilwax-DA, length 15 m, ID 0.53 mm, film thickness 0.1 mm; Varian Chrompack, Bergen op Zoom, The Netherlands) in a Chrompack CP9001 gas chromatograph.

### 2.7. Statistical Analyses

Results are presented as mean ± SEM. Repeated measures ANOVA with Bonferroni correction was used to assess the effect of time and fiber on SCFA and BCFA production. Final NaOH usage was analyzed with one-way ANOVA with Tukey’s *post hoc* test. Significance was *p* ≤ 0.05.

## 3. Results

### 3.1. Sodium Hydroxide Usage

**Figure 1:**
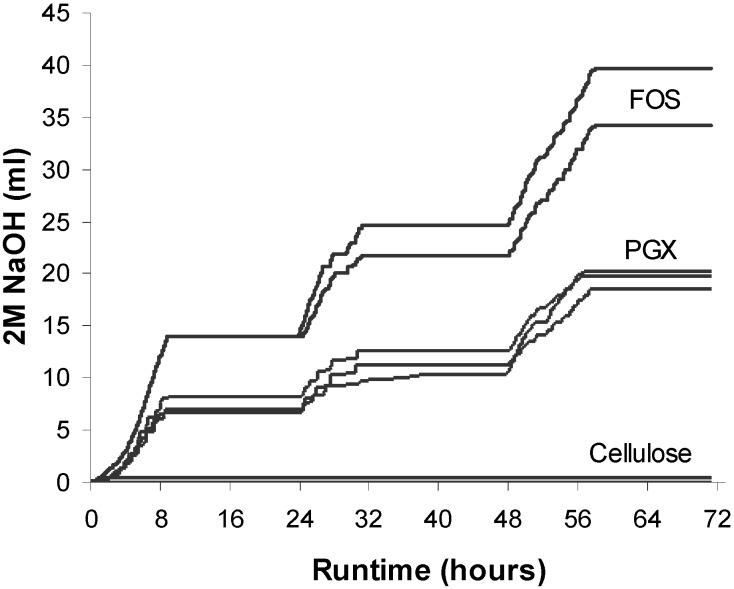
Sodium hydroxide usage during multiple TIM-2 runs over time for fructooligosaccharide (FOS), PolyGlycopleX (PGX) and cellulose. Values at the start of the test period were set to zero. Individual runs are shown where *n* = 2 for FOS and cellulose and *n* = 3 for PGX.

NaOH usage reflects the production of SCFA which, when not corrected, would result in a decrease in pH of the luminal contents as fermentation occurs. The use of NaOH by the TIM-2 system to maintain the pH at 5.8 is shown for FOS, PGX and cellulose in Figure 1. There was no NaOH usage during the test runs with the negative control cellulose indicating that the fiber was not fermented by the microbiota. The positive control FOS showed the highest amount of NaOH usage which reflects the greatest acid production from fermentation of the substrate. The test product PGX^®^ also showed NaOH usage indicating fermentation of the PGX^®^ by the large-intestinal microbiota present in TIM-2. One way ANOVA analysis of the final NaOH usage indicated significantly higher values for FOS (37.0 ± 2.8 mL) than for PGX (19.5 ± 0.5 mL) which was higher than for cellulose (0.3 ± 0.3 mL) (*p* = 0.002). When calculated back to the amount of substrate added to TIM-2, NaOH usage during FOS fermentation was stable over the three day test period. With PGX^®^, NaOH consumption was stable over the first 2 days but showed higher consumption on day 3 in all three separate runs, indicating adaptation of the intestinal microbiota to the substrate and increased ability to ferment the substrate.

### 3.2. SCFA Production

**Figure 2:**
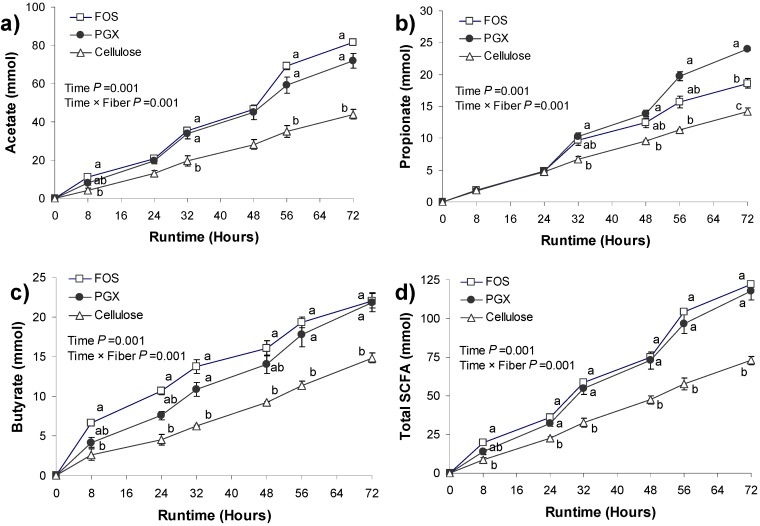
The average cumulative production of (**a**) acetate, (**b**) propionate, (**c**) butyrate and (**d**) total short chain fatty acids (SCFA) in TIM-2, during 72 h on the fructooligosaccharide (FOS), PolyGlycopleX (PGX), or cellulose substrate. Values are mean ± SEM (*n* = 2 for FOS and cellulose and *n* = 3 for PGX). Labeled means without a common letter at the same time point differ, *p* < 0.05. *Y*-axis scales differ across subfigures.

**Figure 3:**
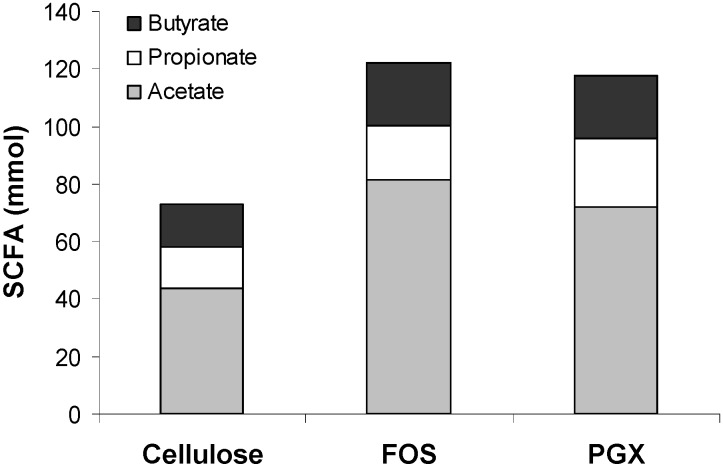
Contribution of acetate, propionate and butyrate to total SCFA production in TIM-2 at 72 h on the fructooligosaccharide (FOS), PolyGlycopleX (PGX) or cellulose substrate. Data are mean values (*n* = 2 for FOS and cellulose and *n* = 3 for PGX).

The amount of SCFA at the start of the test period (0 h) was artificially set to zero. Subsequent values reflect the cumulative production of SCFA after the addition of the test products. There was a significant effect of time (*p* = 0.001) and the interaction of time with the test fiber (*p* = 0.001) for acetate, propionate, butyrate and total SCFA production (Figure 2). At 32, 48 and 72 h, acetate production was higher for FOS and PGX compared to cellulose (*p* < 0.05; Figure 2a). Propionate production was higher for PGX than for cellulose at 32, 48, 56 and 72 h (Figure 2b). FOS was intermediate between PGX and cellulose and at 72 h was significantly higher than cellulose (*p* = 0.001) but lower than PGX (*p* = 0.014). Butyrate production was higher for FOS than for cellulose at all time points during the test period (Figure 2c; *p* < 0.05). PGX was higher than cellulose and not different from FOS, at 32, 56 and 72 h. Total SCFA production was significantly higher for FOS and PGX at all time points from 24 to 72 h (Figure 2d; *p* < 0.05). The contribution of the individual SCFA to total SCFA production at 72 h is depicted in Figure 3.

### 3.3. BCFA Production

As with the SCFA, the amount of BCFA metabolites was set to zero at the start of the test period (0 h). There was a significant effect of time for *iso*-butyrate, *iso*-valerate and total BCFA (*p* = 0.001). There were no significant differences in *iso*-butyrate production between the test fibers (Figure 3A). *Iso*-valerate production was significantly lower for FOS *versus* cellulose but not PGX at 56 h (*p* < 0.05). At 72 h, production of *iso*-valerate was lower for FOS *versus* cellulose and PGX (*p* = 0.005) and PGX was lower than cellulose (*p* = 0.05). Total BCFA production was significantly lower for FOS compared to cellulose at 48, 56 and 72 h (*p* < 0.05) whereas PGX was lower than cellulose at 72 h (*p* = 0.05).

## 4. Discussion

The present study examined the fermentation potential of the novel polysaccharide, PGX^®^, using the *in vitro* TIM-2 system. SCFA have been proposed as mediators of at least some of the beneficial effects of dietary fiber on colonic health, cardiovascular risk factors, and obesity and type 2 diabetes risk [1,11]. We report that total SCFA production from PGX^®^ is similar to the positive control FOS. Compared to FOS, there was lower acetate (NS) and higher propionate production for PGX^®^ (*p* = 0.014) which may explain some of the metabolic effects of PGX^®^ including reducing hypercholesterolemia [17,18,19,20].

The first major finding of this study is that total SCFA production at the end of the 72 h test period was similar between FOS and PGX^®^ and significantly higher than cellulose. While acetate and butyrate production was similar between FOS and PGX^®^, with FOS being slightly higher, the production of propionate with PGX^®^ at the end of the fermentation period was significantly higher than FOS and cellulose. Although the reasons for the generation of distinct SCFA production profiles between PGX^®^ and FOS are not entirely known, it is likely that the glycosidic linkages and monosaccharide composition of the two fibers result in different microorganisms that ferment the substrates. For example, the genome of *Bacteroides thetaiotaomicron* contains numerous (>100) glycosylhydrolases [21] that have been hypothesized to be involved in the breakdown of several host and dietary glycans. While *Bacteroides* species have long been known to produce propionate [22], it is also plausible that other genera besides *Bacteroides* may be involved in fermentation of PGX^®^. The higher production of propionate with PGX^®^ is interesting in light of the proposed satiety-enhancing effects and demonstrated cholesterol-lowering effects of propionate [23]. Propionate has been shown to inhibit hepatic cholesterol synthesis in both humans and animal models [23,24,25]. Propionate may lower serum cholesterol by reducing cholesterol synthesis in the liver, via 3-hydroxy 3-methylglutaryl CoA reductase [26], or enhancing secretion and synthesis of bile acids [27]. These mechanisms may help explain part of the ability of PGX^®^ to lower serum cholesterol in rodents [17,18,19] and humans [20]. In addition, recent *ex vivo* experiments with adipose tissue have shown that propionate beneficially affects cytokine and adipokine secretion by adipose tissue [28,29].

The second major finding is that sodium hydroxide consumption, an indicator of fermentation in the system, increased for PGX^®^ on day 3 compared to the first two days. This pattern of fermentation suggests that the intestinal microbiota adapted to the substrate making them better able to ferment the substrate with increasing time. This period of adaptation is perhaps not surprising in light of evidence in human subjects where changes in serum SCFA in response to dietary fiber intake took many months (*i.e.*, >3 months) to occur [30]. It is likely that at least part of this adaptation period can be attributed to changes in the complex interactions of the bacteria community in the host [31]. Adaptation to PGX^®^ occurred relatively quickly in our *in vitro* system (*i.e.*, by day 3), likely due to the absence of other fermentable carbohydrates. There may be important implications for the period of adaptation in humans consuming the product in that benefits attributable to fermentation *per se* would accumulate over time.

The third major finding is that the production of BCFA, a marker of protein fermentation, was highest for cellulose, lowest for FOS and intermediate for PGX^®^. While SCFA are the principal products of saccharolytic activity (*i.e.*, breakdown of carbohydrates) in the gut by *Bacteroides* spp., *Lactobacillus* spp. and *Bifidobacterium* spp., BCFA are the product of protein degradation carried out by dominant proteolytic bacteria including *Bacteroides* spp. and *Clostridium* spp. [32]. BCFA, chiefly *iso*-butyrate and *iso*-valerate, are markers of bacterial protein fermentation and their presence is associated with the production of toxic metabolites such as ammonia and phenols [10]. The production of BCFA in the TIM-2 system is reflective of the amount of carbohydrate substrate available for the microbiota. Indeed, one of the major mechanisms by which carbohydrates can reduce the production of BCFA is via availability of fermentable carbohydrate substrate [33]. When carbohydrate energy is low in the lumen, bacteria will ferment protein or amino acids to obtain energy [33]. In our system, cellulose is poorly or not fermented [34], as demonstrated by the lack of NaOH consumption in Figure 1 and therefore fails to provide substrate to the gut microbiota. As a result, the fermentation of proteins derived from the Standard Ileal Efflux Medium and bacteria secretions occurs. FOS on the other hand is a highly fermentable substrate, as shown by the greatest NaOH consumption in Figure 1 and thereby supplies abundant carbohydrate substrate.

**Figure 4:**
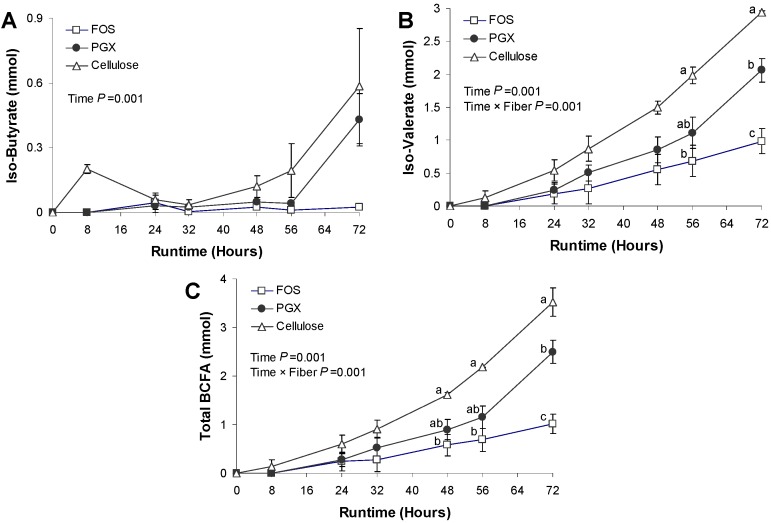
The average cumulative production of (**a**) *iso*-butyrate, (**b**) *iso*-valearate and (**c**) total BCFA in TIM-2 during 72 h on the fructooligosaccharide (FOS), PolyGlycopleX (PGX) or cellulose substrate. Values are mean ± SEM (*n* = 2 for FOS and cellulose and *n* = 3 for PGX). Labeled means without a common letter at the same time point differ, *p* < 0.05. *Y*-axis scales differ across subfigures.

While the lack of NaOH consumption with cellulose (Figure 1) may seem contradictory with the generation of fatty acids seen in Figure 2, the SCFA produced in the experiments with cellulose are in fact exclusively from protein fermentation. Previous work has shown that cellulose is poorly fermented [15] and that the SCFA produced stem from protein fermentation, as also indicated by the increase in ammonia in those experiments [15]. Given that ammonia raises the pH, there is no need for the system to secrete NaOH. Indeed, the pH in the runs with cellulose slowly increased to 6.9 (not shown), rather than staying constant at 5.8. This is due to the production of significant amounts of ammonia which has a pKa of 9.25. Hence, no NaOH secretion was needed to maintain a pH of 5.8, but SCFA production was due to the proteinaceous substrates added with SIEM (Figure 2 and Figure 3), reflected by increased BCFA (Figure 4).

The suppression of BCFA production by FOS supports previous work in which inulin suppressed BCFA and ammonia production in the TIM-2 system [7] and similar results have been obtained with several glucose-based fibers [15]. PGX^®^ was shown to be intermediate in both NaOH consumption and BCFA production. The stimulation of carbohydrate fermentation by the microbiota is beneficial, not only for the production of SCFA as an energy source for the host and particularly colonocytes but the microbiota also provides for a “nitrogen sink”. As saccharolytic bacteria grow they incorporate putrefactive metabolites into cellular proteins and thereby remove proteins and harmful protein metabolites from contact with host tissue [10,35]. In humans, resistant starch and other fermentable substrates such as soluble maize fiber and polydextrose have been shown to reduce the accumulation of potentially harmful metabolites of protein fermentation in the colon [35,36]. PGX^®^ appears to act similarly, albeit to a lesser extent than would be seen with FOS.

Given the rapid absorption of SCFA and the inherent limitations of using faecal concentrations of SCFA to predict their *in situ* production, the TIM-2 system provides a clear advantage for accurately determining the SCFA production from PGX^®^. In future experiments it might be wise to also measure any remaining PGX^®^ after fermentation, e.g., after 24, 48 and 72 h. In addition, there would be added benefit in measuring other fermentation products such as lactate and putrefactive factors such as ammonia which would provide an even better indication of the shift in balance between production of healthy and toxic metabolites. Lactate is produced by many gut microbiota including lactobacilli, bifidobacteria and enterococci [37]. In addition to the acidifying and anti-microbial effect of lactate, certain lactate-utilizing bacteria are able to convert lactate into butyrate, a key gut-protective SCFA [37]. Although not measured here, fermentation of FOS is known to produce lactic acid (lactate) [7] and therefore it is highly likely that the higher NaOH consumption occurring with FOS (Figure 1) is caused by production of lactate.

## 5. Conclusions

In conclusion, this study demonstrated that PGX^®^ is fermented by the microbiota in TIM-2, a representative *in vitro* model of the human large intestine. In addition, it was shown that when administered daily, the microbiota adapted (in composition and/or metabolically) over time with increased ability to ferment the substrate. The greater propionate production with PGX^®^ may explain, at least in part, the consistent cholesterol-lowering properties of PGX^®^ seen in both rodents and humans. Given emerging evidence for the role of gut microbiota and SCFA in regulating inflammation [28,29,30,31,32,33,34,35,36,37,38,39,40], it will be interesting for future work to examine the effects of PGX^®^ on inflammatory markers, particularly in the context of metabolic syndrome.
